# Div-BLAST: Diversification of Sequence Search Results

**DOI:** 10.1371/journal.pone.0115445

**Published:** 2014-12-22

**Authors:** Elif Eser, Tolga Can, Hakan Ferhatosmanoğlu

**Affiliations:** 1 Department of Computer Engineering, Bilkent University, Ankara, Turkey; 2 Department of Computer Engineering, Middle East Technical University, Ankara, Turkey; University of Rome Tor Vergata, Italy

## Abstract

Sequence similarity tools, such as BLAST, seek sequences most similar to a query from a database of sequences. They return results significantly similar to the query sequence and that are typically highly similar to each other. Most sequence analysis tasks in bioinformatics require an exploratory approach, where the initial results guide the user to new searches. However, diversity has not yet been considered an integral component of sequence search tools for this discipline. Some redundancy can be avoided by introducing non-redundancy during database construction, but it is not feasible to dynamically set a level of non-redundancy tailored to a query sequence. We introduce the problem of diverse search and browsing in sequence databases that produce non-redundant results optimized for any given query. We define diversity measures for sequences and propose methods to obtain diverse results extracted from current sequence similarity search tools. We also propose a new measure to evaluate the diversity of a set of sequences that is returned as a result of a sequence similarity query. We evaluate the effectiveness of the proposed methods in post-processing BLAST and PSI-BLAST results. We also assess the functional diversity of the returned results based on available Gene Ontology annotations. Additionally, we include a comparison with a current redundancy elimination tool, CD-HIT. Our experiments show that the proposed methods are able to achieve more diverse yet significant result sets compared to static non-redundancy approaches. In both sequence-based and functional diversity evaluation, the proposed diversification methods significantly outperform original BLAST results and other baselines. A web based tool implementing the proposed methods, Div-BLAST, can be accessed at cedar.cs.bilkent.edu.tr/Div-BLAST

## Introduction

Sequence similarity search is one of the earliest and most commonly employed tools of bioinformatics for molecular biologists. In current sequence search tools, the results retrieved from the database are typically also highly similar to each other. For many bioinformatics tasks, the result set needs to be diversified to produce a subset of results containing sequences well aligned with the query but sufficiently different from each other. This need is apparent in the use of non-redundant databases such as the *nr* database used in BLAST [1] [2]. However, to the best of our knowledge, no sequence similarity search tool incorporates diversity into the search algorithm. Search diversification has been studied in information retrieval, but it has not yet attracted attention in bioinformatics.

Sequence similarity search is an area that would benefit from more diverse results rather than just top-similar results. Identifying all functional domains of a query sequence, which may be comprised of separate homologous domains in different sequences, can only be established by an approach whose main purpose is to cover most of the query sequence rather than finding the most similar sequence. In this paper, we formalize the problem of diversification and investigate methods to post-process results from the commonly employed search tools to remove redundancy from the results and enable exploratory browsing. An example of such searches is to find proteins with different functions but similar enough to the query sequence. Different segments of the primary structure may correspond to different functional domains. Tools such as BLAST incorporate a domain identification step and present the identified domains to the user in addition to the query results. However, domain identification is limited to known, characterized domains and novel domains in the query sequence will be overlooked by this approach. Such novel domains may be shared by some of the database sequences and a diverse search may identify these regions. For this purpose, finding a diverse set of regions with similar segments would be a more appropriate approach than simply investigating the top-similar sequences. With our proposed method, we are also able to control the effect of diversification based on the dissimilarity of biological functions of sequences.

Sequence alignment is utilized to arrange the sequences of DNA, RNA, or amino acid sequences to identify regions of similarity. Global alignment follows a general similarity measure and attempts to align each residue in every sequence using gaps, and local alignment focuses on determining similar sub-regions. Sequence search tools such as BLAST [1] [2] and FASTA [3] seek similar sequences to a given query in large sequence databases. Our proposed approach is applicable to post-process the results of any sequence similarity search tool. However, for our experiments, we focus on BLAST and on one of its popular variants, Position-Specific Iterated BLAST (PSI-BLAST) [2], which seeks locally similar sequences on protein databases by using profiles updated dynamically in iterations.

BLAST compares nucleotide or protein sequences to large sequence databases, calculates the statistical significance of matches and returns the results with attributes such as query coverage, total score, max score, e-value, and maximal identity. Total score is the sum of the scores of all high scoring pairs(HSPs) from the same database sequence [1]. Unlike BLAST, PSI-BLAST profiles are built by considering evolutionary relationships, and using them enables detection of a protein's distant relatives. As diversification is applied for *blastp*, which is original BLAST for protein-protein search, and for PSI-BLAST, it could be possible to use for all versions of BLAST including nucleotide-nucleotide BLAST, *blastn*, and translation BLAST types such as *blastx, tblastx* and *tblastn*. The translation models may compare nucleotides to amino acids or vice versa.

Diversification of search results aims to produce results similar to the query but different from each other. Although there is no prior work on diversification in sequence search, the notions of diversity and novelty are present in the context of information retrieval and recommendation systems. Carbonell and Goldstein [4] were the first to introduce Maximal Marginal Relevance (MMR) for text retrieval and summarization. This method builds a result set by maximizing the query relevance and minimizing the similarity between documents in the result set. It uses a parameter (λ) that specifies the proportions of relevancy and diversity. Jain *et al.* [5] and Haritsa [6] propose a greedy solution for the k-nearest diverse neighbor search for spatial data. Liu and Jagadish [7] employ the idea of clustering to find a solution to the Many-Answers Problem. They propose a tree-based approach to choose one representative from each cluster consisting of diverse results.

Although diversity search has not yet been explicitly investigated in the context of browsing sequence databases, redundancy elimination during database generation may be a viable alternative. One can decrease the redundancy of these databases by a preprocessing procedure. Commonly used protein sequence databases such as UniProtKB, UniProtKB/SwissProt [8], UniParc [9], and UniRef [10] databases have reduced, non-redundant versions. UniProtKB includes two different databases: UniProt/TrEMBL and UniProt/SwissProt. In UniProt/TrEMBL database, for the fully identical, full-length sequences from one species there is one record. UniProtKB/SwissProt is built with different representative sequences for sequences encoded by one gene in one species. UniParc and UniRef databases are comprised of representatives of 100%-identical sequences, regardless of the species. In the UniRef databases, sub-fragments are included as different records from full-length sequences. These databases implicitly remove identical alignment results by eliminating identical sequences or fragments from the databases. This pre-processing is done in design time and is independent of the query sequence. While queries can avoid identical sequences in the results, most still contain results with too much redundancy, as we also illustrate in the experimental section.

In addition to eliminating the redundancy during database generation, one can reduce redundancy by processing the search results. For example, Berman *et al.* [11] propose a redundancy elimination method in which a stretch of a locally aligned region *I* (i.e., interval) is filtered out from the result set if *K* other intervals include *I* and have better scores than *I*. The whole interval is filtered out from the result set. The methods we propose measure diversity at the amino acid or nucleotide level for the whole result set. Therefore, a sub-interval, which contributes to the overall diversity of the results, may be retained unlike in Berman *et al.*'s method. In addition, we seek diversity at the global level in the result set, whereas Berman *et al.* seek diversity at the interval level individually. Besides this study, search methods in the literature that use culling or clustering, such as Pisces, BlastClust and CD-HIT [12] [13] [14], can also be utilized for redundancy elimination. Pisces [12] is a culling tool that selects the first sequence of the input set and eliminates the sequences with higher pairwise similarity than a given identity threshold. As the chosen set is populated, the remaining sequences are compared to all currently selected ones. BlastClust [13] and CD-HIT [14], [15] are based on clustering rather than culling, and can be used to choose representatives of each cluster to obtain a relatively more non-redundant set of given sequences. BlastClust clusters by starting a pairwise comparison between result sequences, while CD-HIT clusters are generated by using short word filtering. CD-HIT builds clusters by controlling common n-gram words among sequences. It skips pairwise alignment for efficiency. Sequences are sorted and the first one is picked as the first cluster representative. If the next sequence is similar to any cluster representative, i.e., higher similarity than a given threshold, it is included in the cluster. Otherwise, a new cluster is created by using the sequence as a seed. CD-HIT requires user inputs such as the weight of words and a cluster similarity threshold. Additionally, CD-HIT may eliminate potentially important diverse alignments because of the restriction to choose a single representative from each cluster. CD-HIT does not consider diversity at the amino acid or nucleotide level.

To diversify sequence search results, we present two novel methods, BitDiversity and EntropyDiversity, which iteratively construct a set of results that are diversely aligned with the query sequence. We propose a novel diversity measure based on Rao's quadratic entropy [16] to evaluate the result quality. We also evaluate the diversity of the protein functions using a molecular functional ontology subset of the Gene Ontology (GO) [17] terms. For each evaluation, we also test the result significance with Wilcoxon signed rank test [18], which is a non-parametric statistical hypothesis test for two sets of samples. We compare the results of both diversity methods with original BLAST results. We also compare our approach with an adaptation of the CD-HIT method for redundancy elimination. Additionally, we give the query coverage comparison results of diversified sets and the original set. One of the aims of diversification is to find diverse regions of queries, and the diversified set achieves a complete coverage more rapidly than the original set. We provide the significance of the coverage results with Wilcoxon test.

We built an online diverse sequence search tool called Div-BLAST that supports queries using BLAST web services. The tool ranks the result set according to a desired diversity level. As Div-BLAST is a post-processing tool on BLAST, it asks parameters such as database, program, query, etc. Div-BLAST users can choose one of the developed diversity algorithms and the diversity rate. Although our diversification methods need no parameters, we added a feature to allow users to observe similarity and diversity tradeoffs: user may re-rank the results again if they wish. Div-BLAST records the old queries with a unique ID, gives permission to download the result set, and enables users to arrange the results in ascending or descending order with respect to score, e-value, coverage, etc.

## Methods

### Diversification of Sequence Search Results

We aim to diversify sequences from the result set of a query searched using a sequence search tool, e.g., BLAST. One can set an optional parameter *k* to retrieve the top results in a more diverse fashion. We build algorithms that are incremental and do not depend on the value. In other words, the first *k* diversified results are the same as those in the diversified set with *k* = *k+1.* The algorithms may run as re-ranking the result set regarding diversity. The *k* parameter provides speed; it is not necessary to wait for all sequences to be ranked. We expect the *k* diverse sequences to have different alignments from each other with respect to the query. In other words, we want to choose *k* different results that have query coverage on different sections of the given query or different residues within the same alignment region. We present methods for systematizing the diversification problem. In accordance with the above-mentioned diversity definition, in our approaches, we deal not with full-length result sequences but with aligned fragments within the query. In the rest of the paper, the term *result sequence* refers to an aligned fragment.

Eq. 1 represents the general formulation of diversity for our approaches, namely BitDiversity and EntropyDiversity. Both of these approaches are iterative, i.e., in each iteration the sequence that provides maximum difference is added into the current diverse set regardless of the original order in the result set. We initialize the diverse set with the first sequence of the original result set. We fix the length of all result sequences to that of the query enlarged with the gaps formed in the alignments of the query and any result sequence. The algorithm stops when the size of the current diverse set reaches *k*. The proposed BitDiversity and EntropyDiversity methods are explained in detail in the following sections. Briefly, BitDiversity is based on the average of the differences between the candidate and each result sequence, whereas EntropyDiversity considers the general entropy of result sequences and candidates together. The proposed algorithms are executed as a post-processing of the search results, which involves aligned sections of the result sequences.
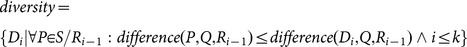
(1)


Here,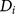 is a result sequence included by the diversified set 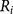, which is a subset of all result sequences 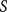. 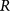
is the top-k diversified set, therefore it equals to 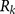. 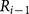
is the chosen diverse set before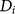. 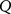 represents the query used in *difference* formula characterized by the diversification methods. Note that 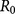 is an empty set.

**Figure 1:**
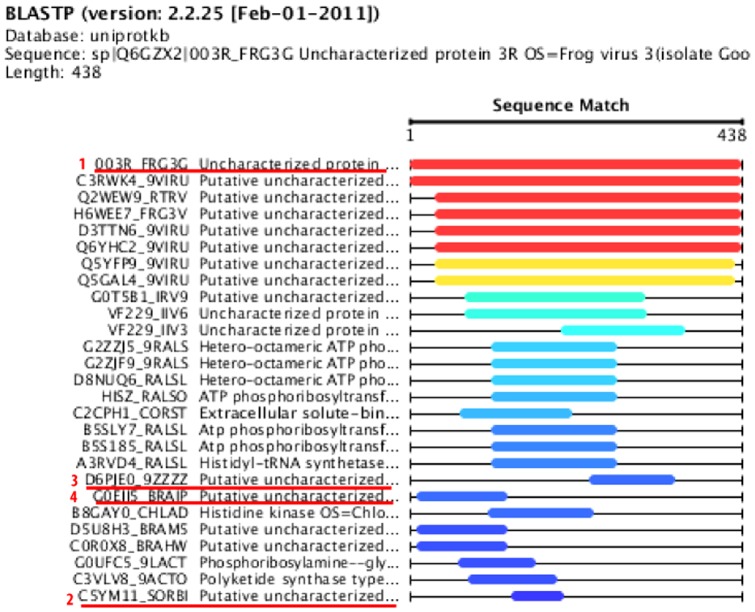
A result set obtained from a BLAST sequence search. Underlined sequences are chosen as top-4 diverse results and the red numbers next to sequences represent the inclusion order into the diversified set.

To illustrate the diversity problem, we provide an example BLAST run. In the example, BLAST returns 27 different sequences, as shown in Fig. 1. The result set contains aligned fragments to the query. We run our pairwise bit comparison diversity method on this fragment set. Fig. 1 shows the diversified set with top-4 diverse sequences, which are underlined in red with the new order after diversification. The diverse results are determined as follows: The diversification algorithm starts with the first sequence in the original result set. The last sequence in the result set is chosen next, which is the most distant sequence to the current result set. The sequence with number 3 is selected as the third diverse sequence because it has no intersection with the second element and has the least overlap (due to its length) with the first one. Last, the forth one is inserted in the diversified set due to no overlap with the second and third sequences in the set.

### Pairwise Bit Comparison

Algorithm 1 shown at Table 1 presents our greedy heuristic that selects a sequence from the initial result set 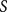 in each iteration, and constructs the diverse *k* results after *k* iterations. In every iteration, the algorithm scans the whole unselected result list. In a sub-iteration, there is also a loop that finds the difference between the candidate result and each sequence in the current diversified result set. In the BitDiversity approach, sequences are treated simply like bit sequences. The aligned residues of a result sequence with respect to query are marked as 1, otherwise as 0. It means every sequence is also represented as a d-dimensional binary vector of 1 or 0, referring to matched or unmatched residues. BitDiversity uses the bit sequences for calculating the difference of two sequences. Here the difference is calculated with the XOR operation, which is a bitwise operator that makes the result bit 0 if a matching occurs; otherwise, the result bit is 1 (Eq. 2):
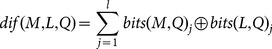
(2)


**Table 1:** DiversitySearch algorithm for a given similar sequence set

	**Inputs**: *S is the original result set, k is the length of diversified subset from the initial result set and Q is the searched query.* **Output**: *chosenList is the diversified subset.*
1:	**procedure** DivSearch (*S*,k,*Q*)
2:	Initialize m as 1//*is the counter for the chosen list*
3:	Initialize divArr//*used for finding the greatest diversity rate*
4:	Initialize notChosenList with *S* (all results except the first)
5:	Initialize chosenList with {the first sequence of *S*}
6:	**Do**
7:	set divArr {}
8:	**for** i **in** 1: notChosenListLength
9:	divArr[i] = Div(notChosenList[i],chosenList, *Q*)
10:	**Endfor**
11:	find j as the index of max valued divArr[i]
12:	add chosenList notChosenList[j]
13:	remove notChosenList[j]
14:	m++
15:	**while**(m< = k)
16:	**End procedure**

The output of the algorithm is a diversified subset(with *k* elements) of the given sequences.

where *l* is the length of sequence, *bits* converts result sequences 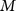
and 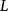, to bitwise sequences with respect to query *Q*. The formula aggregates the XOR results for each position *j* in 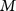
and 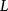.

The total number of 1 s after the XOR operation is considered as a simple diversity measure of two given sequences. Diversity between a sequence and a set of sequences can be defined with various patterns, such as linkage computations [19]. In single and complete linkage approaches, the diversity-relevance measure between a sequence *s* and a sequence set 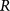 depends on the difference between the sequence and the most similar (single linkage) or most diverse (complete linkage) sequence from the sequence set. The minimum or maximum pairwise difference between *s* and the sequence of 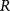 specifies the diversity, depending on single or complete linkage algorithms, respectively. When the difference between *s* and 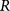 is based on the average linkage method, the average of each difference between *s* and the sequence of 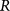 is used for diversity. We experimentally observed that the average linkage approach provides the best results.

The *Div* function at Step 9 in Algorithm 1 (Table 1) depends on the diversity approach used. For BitDiversity, it calculates the diversity rate based on the average diversity rate of the current candidate sequence and each sequence in the current chosen result set.

### Entropy-Based Diversity

Entropy has been used for measuring diversity in information retrieval [20]. In the context of bioinformatics, it is applied to evaluate the quality of multiple sequence alignment, but with the opposite goal of having low entropy, i.e., to achieve a high-quality alignment [21]. We follow a similar idea for a sequence similarity search, where the multiple alignment of the result set is readily available in the form of a star alignment, where the center sequence is the query sequence. While the result set is similar to the query, a diverse result set implies a low-scoring multiple sequence alignment. Therefore, we aim to have a high entropy score in the result for diversity. We propose an entropy-based approach, EntropyDiversity, that chooses the *n^th^* sequence from the result set depending on the entropy of the chosen sequences and the candidate sequence together, and finds the candidate sequence that makes the entropy highest. Entropy is defined as:
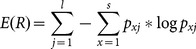
(3)


where 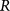 is a result set, *l* is the length of the sequence, *s* is the size of the letter set, *x* represents the elements of the given alphabet (the alphabet, in other words, the letter set, could be comprised of 20 amino acid letters or 0 and 1), and *p_xj_* is the probability of *x* in the *j^th^* tuple of all *m* sequences (*m* is the size of result set.)

**Figure 2:**
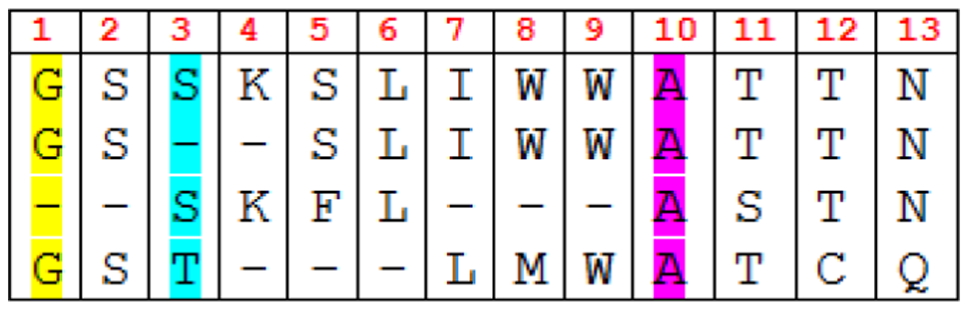
A result set to explain entropy based diversity on amino acid level.

Fig. 2 illustrates an example of entropy-based diversity. In the illustration, 13 is the length of sequences, which is represented by *l*. In the amino acid set (*s = 20*), let us assume the indices of G, S, A and gap(-) are 1, 2, 3 and 4, respectively. For *j = 1*(first column) *x = 1*(refers to G amino acids) *p_1,1_* will be 0.75 (three Gs out of four residues) and *p_4,1_* will be 0.25. Similarly *p_2,3_*, the probability of Ss in the third column, and *p_3,10_*, the probability of As for the tenth column, are 0.5 and 1.0, respectively.

In EntropyDiversity, one can look at either the entropy of amino acid residues or the bitwise entropy, which deals with whether the piece of sequence is aligned. For the former, the alphabet size is 20 (possible amino acids) and for the latter it is 2 (0 and 1). At Step 9 in Algorithm 1, we design the function *Div* as the combination of the amino acid and bitwise entropies by taking their average to utilize them both. To balance between amino acid-based and bitwise entropy, we normalize both of them before averaging. In normalization, Eq. 4 is used as the maximum value of the entropy.
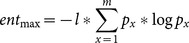





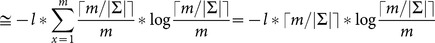
(4)


where 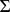 is the alphabet, *l* is the length of the candidate sequence, and *m* is the length of the multiple alignment of the result set.

We note that for both methods, BitDiversity and EntropyDiversity, no user-defined parameters are required. As we post-process the results of similarity search, the sequences in the raw result set are already similar to the query. Hence, we focus on diversifying the results.

### Measures for Evaluating Diversity

#### Sequence Diversity Measure

We first propose a measure to evaluate the diversity of a sequence set that consists of result sequences already aligned with the query. We adapt a version of Rao's quadratic entropy [16], [22] (which was initially used for diversity of/within populations) as the basis of this new measure. Quadratic entropy is used for non-discrete instances; it takes distances into account. Eq. 5 shows the basic quadratic entropy formula:
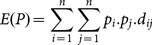
(5)


where 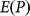 is the entropy of the whole set (for all instances 1 to *n*), *p_i_* represents the probability of the *i* ^th^ instance and *d_ij_* is the distance between the *i* ^th^ and *j* ^th^ instances.

To compute entropy as in Eq. 5, a dissimilarity matrix is needed. To convert the amino acid substitution matrices, which incorporate similarities, into dissimilarity matrices, we apply Eq. 6 to each element in the BLOSUM62 [23] matrix and use it as the distance matrix for the entropy calculations. In addition to the existing rows and columns of the original BLOSUM62 matrix, we add a new row and a column for the non-aligned symbol to the query. Note that; with the new values for the matrix, we obtain a dissimilarity matrix with 0 diagonal.
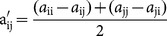
(6)


In Eq. 6, a′*_ij_* is the new value for element a*_ij_*. a*_ij_* and a*_ji_* represent the raw distance between the *i^th^* and *j^th^* elements. Since a*_ij_* and a*_ji_* are different, the new distance values are not symmetric. To obtain a symmetric distance matrix, we use the average of the new raw distance values in Eq. 6.

The diversity of a sequence of length *l* is computed as in Eq. 7. After the result sequences are multiply aligned with respect to the query, for each tuple we calculate the quadratic entropy with the new dissimilarity matrix. The average of the entropy of the tuples is the diversity rate of the given sequence set.
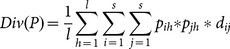
(7)


In Eq. 7, *l* is the length of the sequence and *s* is the size of the letter set (including the amino acids and the gap and non-aligned part symbols). *p_ih_* and *p_jh_* are the probability of the *i* ^th^ and *j* ^th^ letters for the *h^th^* position of all *m* sequences (*m* is the size of the result set). The probability depends on the frequency of the given position. Note that if the letter does not exist in the position, the probability is 0; additionally, for the same letter, the entropy is also 0 since d*_ij_* equals zero. For Rao's entropy of the given elements having positive distance values, the lower bound is 0 when all objects in the set are the same (the distance is 0.) However the upper bound is not generic and it is the maximum distance among the element pairs. In this case, the upper bound is 19, which is the maximum value in the distance matrix based on BLOSUM62 (by using the formula in Eq. 6).

Note that the dissimilarity matrix also includes the unmatched residues with respect to the query. It could be considered as a gap; however, it should be more distant from the amino acids than the gap symbol (a gap is created during alignment.) Hence, the “non-aligned” symbol is assigned with a value twice the value for the gap. In our experiments, this heuristic produces satisfactory results. Thanks to this process, we preserve the importance of “differently aligned sections” while also taking amino acid-based assessment into consideration. In other words, we are looking at the variety of matching and unmatching sequence parts with respect to the query by considering the relationship between amino acids.

We use the above-mentioned measure to evaluate the quality of the results returned by different diversification approaches. While the same measure can be used within the proposed diversification algorithms, we choose to follow simpler measures for reduced computation complexity, hence for more efficient browsing. The performance evaluation does not have the time restrictions of an online search. Our experiments also confirm that the proposed methods do not improve significantly even when such a complex diversity measure is used for diversification. However, taking conservative substitutions into account may be important to signify common evolutionary changes in the level of amino acids. The scores of BLOSUM matrices intuitively give an insight about such substitutions, and we use the BLOSUM-based distances in this algorithm. In addition, taking conservative substitution into account will not affect the ordering of the results when such substitutions are observed in all result sequences. Therefore, there will be no significant difference between methods that consider conservative substitution and methods that do not.

#### Functional Diversity Measure

To check whether diversification methods also provide functional diversity, we propose a functional diversity measure based on GO annotations of proteins in the result set. It has been shown that due to divergent or convergent evolution of protein functions, similar sequences may exhibit different functions [24]. In divergent evolution, the same ancestor often generates super-families of functional proteins catalyzing a diversity of reactions. Conversely, in convergent evolution of functional proteins, the proteins which catalyze the same reaction are independent of each other [25]. Although these conditions are valid for many proteins, controlling functional diversity over result sets would still give insights about the importance of sequential diversity. Another aim of diversity in the primary structure of sequences may be to obtain proteins with different functions.

To compute the functional dissimilarity of a set of protein sequences, we utilize known functions of proteins. As functional information, we use the GO terms [17] belonging to the molecular function ontology. Gene ontology comprises three ontologies: biological process, cellular component, and molecular function. The ontologies are presented as directed acyclic graphs (DAGs) in which the terms form nodes and the two kinds of semantic relations (‘is-a’ and ‘part-of ’) form edges.

For the similarity of functions in the molecular functional ontology, we use Wang *et al*.'s semantic similarity [26]. The authors proposed a method to compute a GO term's semantics into a numeric value by aggregating the semantic contributions of their ancestor terms in the GO graph, and use the values to measure the semantic similarity of GO terms. They consider the similarity of terms not only based on their distance by using the closest ancestor, but also the specificity (depth in the GO graph) of the terms. Hence, the terms that are children of a parent (siblings) close to the root of the ontology do not have the same similarity as siblings that are close to leaf nodes. While Eq. 8 shows semantic value of a GO term, Eq. 9 represents the semantic similarity of two GO terms. The similarity between genes and proteins are computed by considering the pairwise semantic values of the sequences' common and divergent GO terms.
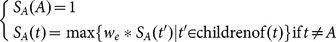
(8)


In Eq. 8, *t* refers to the terms related to *A* GO term, which means all *t*s are included in DAG_A_, the partition of the full DAG comprised of *A* and its ancestors. Note that there are two different weights (*w_e_*) for each semantic relation (‘is-a’ and ‘part-of ’); however, for the molecular function aspect of the GO DAG, the 'part of' relation does not exist.
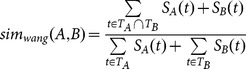
(9)


**Figure 3:**
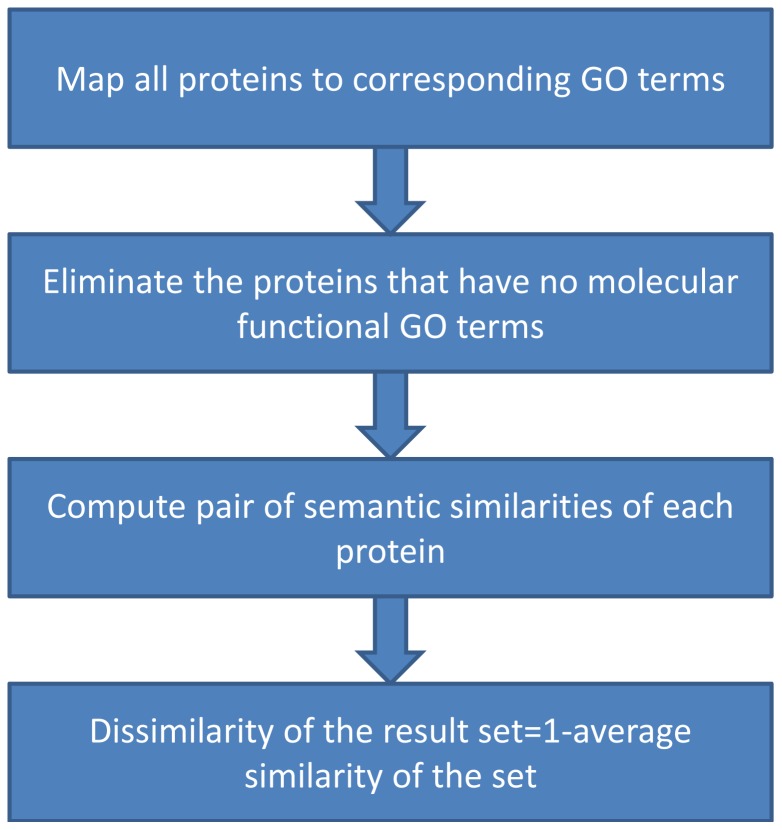
The steps for finding the functional diversity of a set of protein sequences.

Fig. 3 shows the steps of finding the dissimilarity of a result set. The proteins (in the result set) having EBI GO annotations are included for the dissimilarity measure. The dissimilarity is defined as 1-Wang's similarity, whose range is 0 to 1.

## Experiments

### Div-BLAST Design and Implementation

Div-BLAST is a web based service that searches the primary structure of biological sequences with a BLAST-like interface. Basically, the program executes a similarity search for a given sequence in a chosen database and diversifies the results based on the proposed diversification algorithms. Similar to BLAST, it uses search parameters such as database, program, query etc., and diversity parameters of method and rate. Div-BLAST includes a property to observe the tradeoff between the diversified results and the original search results. The user may also change the rate after retrieving the search results. Div-BLAST also saves the searched queries with a unique ID, gives permission to download the result set as txt file, and helps users sort the results in ascending or descending manner with respect to score, e-value, coverage, etc. The user can choose to receive information about the query by an email, and keep her own search history.

Div-BLAST utilizes EBI-EMBL Web Services [27] instead of searching databases on the server. After the web services send the output of the query to the server, the order of the initial search set is arranged according to the chosen diversity method. In the background, we parse xml and txt files retrieved from BLAST services. For the tool implementation, we used Java, JavaScript and HTML. Additionally, we employed ZK [28], which is an open-source Ajax-based web framework integrated into Java. The source code is given as an open-source project and requires Apache servers while running locally. Our web application is compatible with all browsers. The results are gathered from BLAST web services, so it has to wait its execution. We currently utilize a modest server and plan to upgrade as the tool becomes more popular. As we also provide the source code, others can improve the tool and provide their own web service.

### Dataset

We used different sets of sequences, OXBench [29] and UniRef50 [10], for querying on UniProtKB, UniProt/SwissProtKB [8] and UniRef50 databases in PSI-BLAST and BLAST. Our first query set is OXBench data, which is a benchmark for multiple sequence alignment evaluation. OXBench data set is based on 3Dee [30] database of protein structural domains. It has three sub-datasets: master, extended and full. The master set comprises 672 domain families whose three-dimensional structures are known. The extended set is populated with high-scoring homologous sequences of the master set. The two sets mentioned above only include domain sequences, while the full set incorporates full-length protein sequences including the domains. There are 605 different families in the set. As query, we use the full set in our experiments with one representative from each family. The sequence lengths range between 45 and 1124 in the full dataset. The average sequence length is 307. This set is important because it includes sequences with single, double and more domain sequences.

For the second set of experiments, we built a data set using 1000 UniRef50 sequences of different lengths. The set is used as the query set for the sequence search in PSI-BLAST, which is more successful in finding remote homologs with a higher speed [31] rather than BLAST. UniRef is a non-redundant database with different threshold values: 100%, 90% and 50%. Initially, UniRef100 was created to supply non-overlapping sequence sets by combining identical sequences and sequence fragments. UniRef90 and UniRef50 are built upon the UniRef100 database. Each cluster contains sequences that have at least 90% or 50% sequence identity to the longest sequence, respectively. For this dataset, there is no prior information about the chosen sequences, such as their domains. The main purpose of this dataset is to observe diversification performance in a random dataset. The shortest sequence of this set has 12 amino acids, while longest one has 5287. The average sequence length is 421.

### Setup

We analyze query results performed in three different databases: UniProtKB, UniProtKB/SwissProt, and UniRef50. The last two databases are commonly used non-redundant databases and the first one has both reviewed and unreviewed sequences together. UniProtKB includes 30,309,136 sequences. UniProKB/SwissProt contains 539,165 sequence entries and UniRef50 consists of 21,824,511 sequences. We performed the experiments with both the *psi-blast* and *blastp* tools. As mentioned above, experiments were done in two different sets. Note that the first experiments used OXBench and the second used UniRef50.

We evaluated the proposed diversification algorithms, BitDiversity and EntropyDiversity, by comparing them with the original results of the BLAST modules on both datasets. We also compared our algorithms with an approach that reduces the result set using the CD-HIT tool. This tool enables clustering with different setups, including similarity level and word sizes. We set three different similarity thresholds and word lengths: 90% similarity with quadrigram words, 70% with trigram words, and 40% similarity with bigram words. Our aim was not to control the word size effect, however, CD-HIT algorithms require decreasing word length while reducing similarity threshold. For these thresholds, the optimum length of the words were given. We used the sequences aligned to the query as the CD-HIT input set. The non-aligned residues are indicated with an '*X'*, which is a placeholder for unknown or insignificant amino acids. Only the fragments of result sequences are considered, because we measure diversity on only aligned regions to find differently aligned sequences. On the other hand, when clustering or any other diversification methods are applied to a set that includes whole sequences of genes or proteins, one such sequence that may pass the threshold may not be diversified with respect to a given query.

## Results

**Figure 4:**
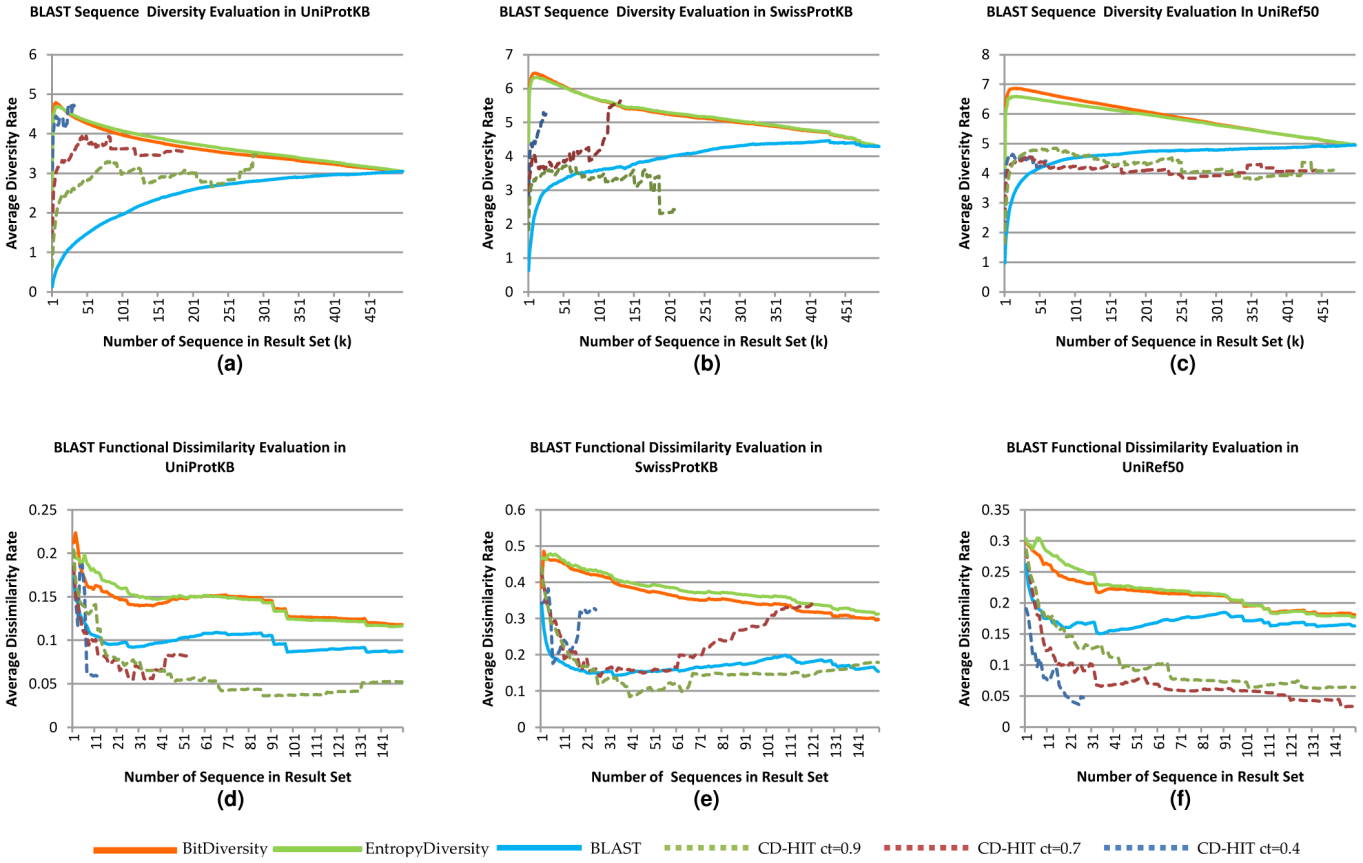
Comparisons based on sequence diversity and functional dissimilarity measures in different databases: UniProtKB, SwissProtKB and UniRef50. The experiments are done by using OXBench dataset on BLAST Web services. For a, b, and c, the x axes represent the average sequence diversity rate calculated with Rao's entropy method and the y axes show the size of the diversified set(k). For d, e, and f, the x axes represent the average functional dissimilarity rate based on Wang et al.'s similarity on molecular function GO DAG. The y axis is the same as the a, b, and c.

**Figure 5:**
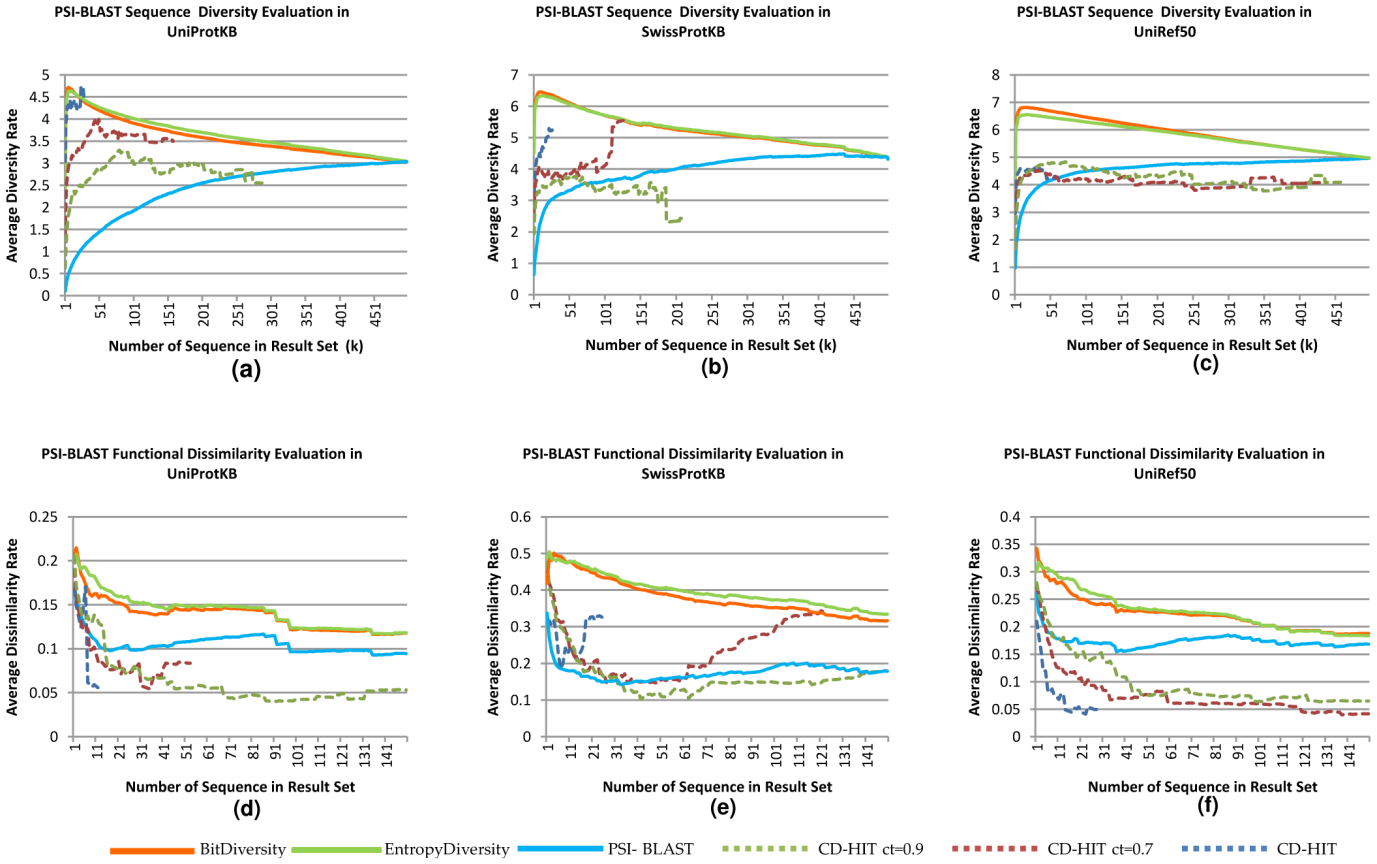
Comparisons based on sequence diversity and functional dissimilarity measures in different databases: UniProtKB, SwissProtKB and UniRef50. The experiments are done by using OXBench dataset on PSI-BLAST Web services. For a, b, and c, the x axes represent the average sequence diversity rate calculated with Rao's entropy method and the y axes show the size of the diversified set(k). For d, e, and f, the x axes represent the average functional dissimilarity rate based on Wang et al.'s similarity on molecular function GO DAG. The y axis is the same as the a, b, and c.

**Figure 6:**
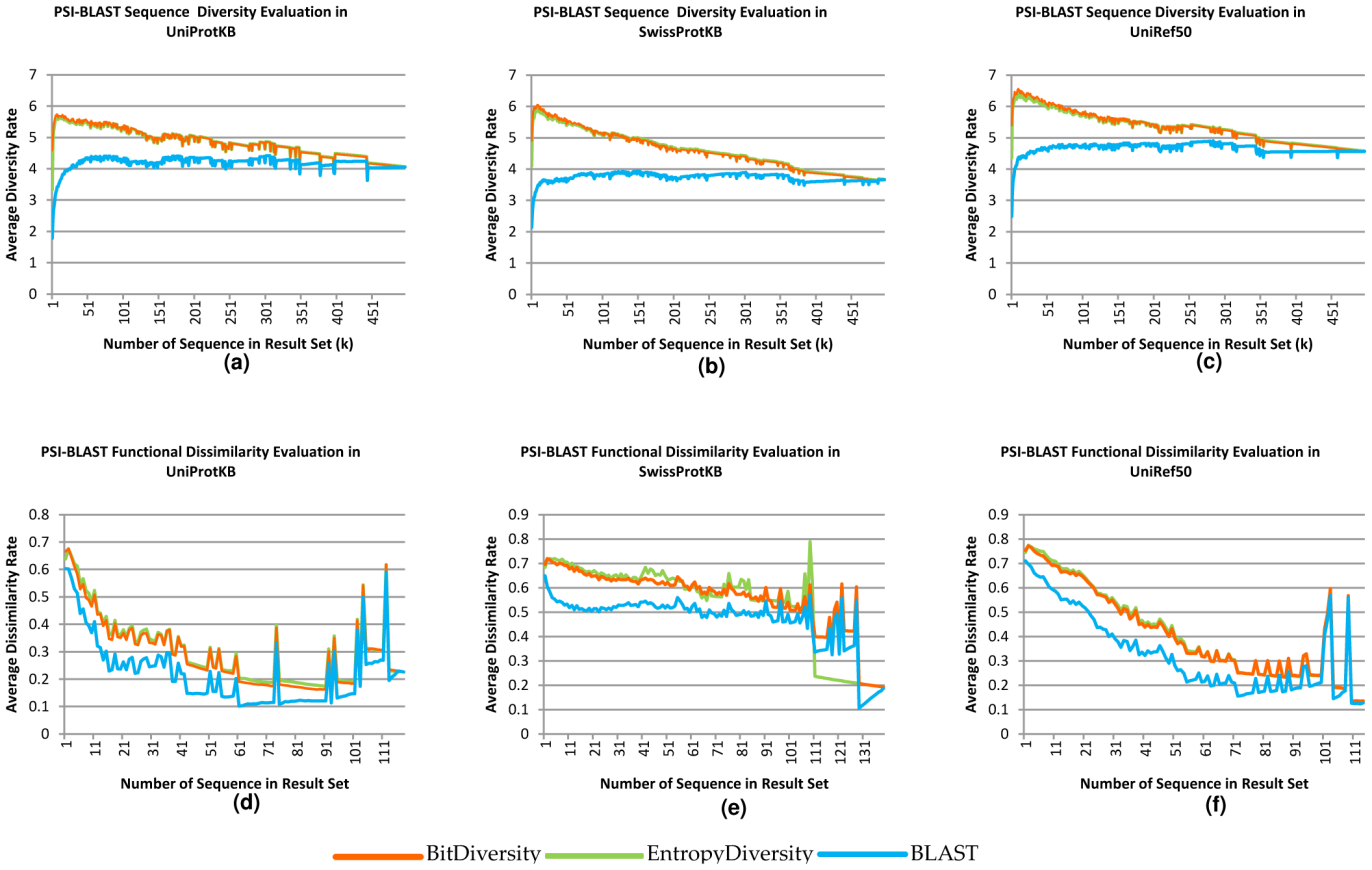
Comparisons based on sequence diversity and functional dissimilarity measures in different databases: UniProtKB, SwissProtKB and UniRef50. The experiments are done by using random 1000 UniRef50 sequences on PSI-BLAST Web services. For a, b, and c, the x axes represent the average sequence diversity rate calculated with Rao's entropy method and the y axes show the size of the diversified set(k). For d, e, and f, the x axes represent the average functional dissimilarity rate based on Wang et al.'s similarity on molecular function GO DAG. The y axis is the same as the a, b, and c.

The evaluation results are illustrated in Figs. 4, 5, and 6. The first two figs. (4 and 5) are related to the first set of experiments, while Fig. 6 shows the results of the second set. In the figs., for each possible *k* (less than or equal to the result set size), we plot the average diversity rate of the diversified result sets with *k* sequences. For example, for *k* = 35, we plot the average of the diversity rates of the result sets with at least 35 sequences. We also plot the diversity rates of the original BLAST and PSI-BLAST result set to compare them with our methods. Note that as the non-redundancy rate increases (UniRef50> Swiss-Prot> UniProtKB), the diversity rates improve better in the original BLAST and PSI-BLAST result sets in both experiments (Figs. 4-a, b, c; 5-a, b, c and 6-a, b, c). This is not surprising because there is significant pre-processing in the preparation of these databases. However, our methods are online and do not rely on this preprocessing, and still work considerably well even with redundant data.

### 

#### Sequence-Based Diversity

For the first evaluation, sequence-based diversity using quadratic entropy, all experiments with different databases show that our results obtained with both the entropic and pairwise methods are significantly more diverse than the BLAST and PSI-BLAST results (Figs. 4-a, b, c; 5-a, b, c and 6-a, b, c). We test the statistical significance of our methods and the BLAST/PSI-BLAST with Wilcoxon signed rank test. We use **τ = 0.05** as the significance threshold. In all databases, the test gives extremely small *p*-values (∼0) for each *k* diversified result set up to *k*≈400. In the OXBench dataset, the difference is significant up to *k*≈500. The results of the proposed methods are close to each other, as evident by looking at the averages on both datasets and on both BLAST modules.

In the comparisons with CD-HIT, our algorithms achieve significantly better diversity on sets of results up to *k*≈15, *k*≈100, and *k*≈170, where cluster similarity thresholds for CD-HIT are set to 0.4, 0.7, and 0.9, respectively. CD-HIT clusters are built by looking at the similarities within a given set. Therefore, the number of clusters is not known before clustering, which means that one may not get a result set with the desired number of sequences; it may return fewer results than expected. In the CD-HIT program, as the similarity threshold within clusters increases, the number of clusters decreases as observed in Figs. 4-a, b, c, and 5-a, b, c.

As expected, the diversity rates have a decreasing trend while the instance number increases because the methods first try to choose the most different sequences. There are minor fluctuations because of the independency of the evaluation criteria and the methods' diversity criteria. In comparison, the diversity levels of the BLAST and PSI-BLAST results increase while the instance number decreases because more similar results to the query are obtained at the beginning.

In addition to finding mean values for each *k*, we also analyzed the standard deviation related to given mean values. The results show that except in the first experiment with UniProtKB, the BLAST/PSI-BLAST results have more deviation than the diversity methods. The averages of the deviations are 1.4 for EntropyDiversity, 1.43 for BitDiversity and 1.47 for the PSI-BLAST results in UniProtKB. These values in SwissProt are 1.32, 1.35 and 1.38; for UniRef50 they are 0.95, 0.98, and 1.01, respectively. The difference between diversity methods and the BLAST/PSI-BLAST is statistically significant according to Wilcoxon's test (*p*<0.05).

#### Functional Diversity

While sequential diversity is different from functional diversity, they both provide useful insights about different aspects of sequences. The functional dissimilarity rates of the result sets are illustrated in figs. 4-d, e, f; 5-d, e, f and 6-d,e, f. In the graphs, the maximum instance number is lower than the original result set size, because not all sequences are annotated with GO terms and we do not compute functional dissimilarity for all the result sets due to the long running times. In the experiments with OXBench sequences, we choose 100 random sequences out of 605, and compare sets (original PSI-BLAST/BLAST and CD-HIT methods) up to 150 sequences. The experiments show that our methods produce more diversity than all the alternatives, including CD-HIT, with statistical significance (*p*<0.05). While CD-HIT performs better than BLAST and PSI-BLAST on sequential diversity, it performs poorly on functional dissimilarity. A reason for this is that sequences with short functional regions are likely to be eliminated by CD-HIT due to overall similarity. The tools we propose signify local diversity; hence, they are also able to provide more functionally diverse results.

In the second experiment, we choose 250 query results with approximately 150 as the maximum size of result set to perform a complete comparison for each sequence and avoid the heavy computational load with larger *k*s. In evaluating this measure, we see that as the number of proteins increases, the required computations also greatly increase. Because, even computing the semantic similarity of two proteins requires computing the pairwise similarity between all GO terms and their ancestors. The measure depends on pairwise protein similarities to calculate an overall dissimilarity within a set of proteins. To avoid long running times, we performed this evaluation for the first 150 query results. Here, the maximum number of GO annotated sequences are 116, 140, and 113 for UniProtKB, SwissProt and UniRef50 databases, respectively. As seen in 6-d, e, and f, our methods give more diverse results than PSI-BLAST with respect to the functional dissimilarity measure. According to Wilcoxon signed rank test, the difference between the proposed diversification methods and PSI-BLAST is significant (*p*<0.05), especially for the first half of the *k* values. Except for the results of UniProtKB database, the difference between the original set and others is significant up to *k*≈100. However, for the functional dissimilarity evaluation, we did not find a consistent difference between diversification methods.

**Figure 7:**
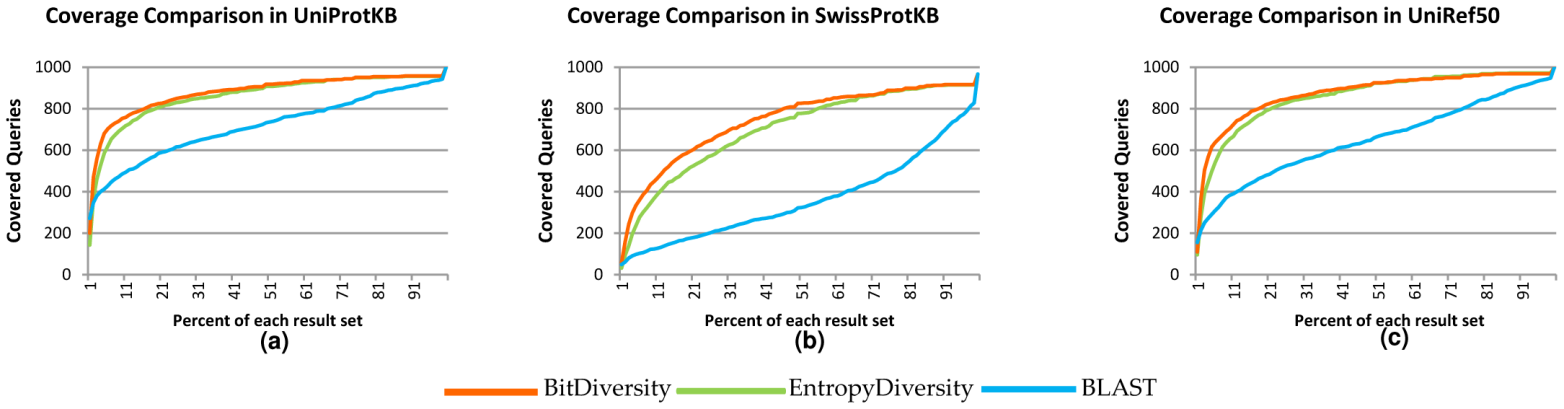
Coverage comparison graphs between original BLAST and diversity based approaches result sets in different databases: UniProtKB, SwissProtKB and UniRef50. The x axes represent the number of result sets that cover all sections of the given queries. The y axes show how much percent of a result set is used.

In the last set of experiments, we analyze the query coverage of result sequences in the second dataset. In our experiments, we have full coverage, i.e., every residue of a query is included in one or more result sequences, on 479, 858 and 508 queries out of 1000 random queries in the UniProtKB, UniProt/Swiss-Prot and UniRef50 databases, respectively. Since the UniProtKB database includes redundant sequences, the result set may contain highly similar sequences. The UniRef50database is smaller compared to the others; hence, the number of fully covered queries in UniRef50ismuch smaller compared to SwissProt. In the UniProtKB database, BitDiversity achieves the full coverage with just 3% of the result set, while EntropyDiversity does the same with 4.5%. PSI-BLAST needs 7.5% of the result set on average to reach full coverage. The rates for the SwissProt database are 1%, 1.5%, and 4.5%, respectively. For the UniRef50 database, 3%, 4%, and 10% of the results achieve full coverage. Note that while investigating coverage, we do not include the first result sequences, which are the same sequences as the queries. This method may not always be observed; however, in our experiments we use known sequences, and the first result is always the query itself. Fig. 7 shows the relation between the number of sequences in the result set and the query coverage. The fig. also includes non-covered query results; the maximum coverage is considered full coverage for a query.

Figs. 7-a, b, c show that the diversification methods have more sequences covered in the same percentage of a result set. Since the size of result sets for each query may be different, we use the percentage of the result sets. We obtain significant p-values (less than τ = 0.05) with Wilcoxon's signed-rank test between the diversified and original results.

## Conclusions

Diverse browsing of sequences and structures is essential for exploratory research in bioinformatics. The current approach of curating non-redundant databases and eliminating identical sequences or fragments is costly and prone to error. In addition, as we illustrated in the experimental section, most queries still contain results with too much redundancy. Alignment and search tools need to perform diversifications tailored for each query. To the best of our knowledge, ours is the first work that investigates diversity in sequence search and alignment. We propose quality measures and methods to diversify the results of sequence similarity search tools. As the result set already includes top-matching sequences, we focus on selecting a diverse subset of this result. To obtain non-redundant results, one could either specify a similarity threshold and omit the sequences that have more similarity than the threshold, or use clustering algorithms. However, these approaches would fail to return enough results and may not supply the desired diversity regarding the query. To overcome this problem, we first presented a pairwise bit comparison approach, BitDiversity, by treating the sequence matches as bit sequences. BitDiversity stresses the diversity in matching locations without considering amino acid differences in those locations. Diversity rate is calculated with the XOR operation for the bit sequences of two sequences. We propose another approach based on entropy and also focused on diversification at the amino acid level. In EntropyDiversity, we compute the entropy of each *i* ^th^ position(*i* is from 1 to the size of the multiple alignment of all result sequences with respect to the query), and in each iteration the sequence that maximizes entropy is chosen to be added to the result set. For both proposed approaches, we investigate design alternatives for calculating the difference between two sequences, and choose the appropriate ones.

To evaluate the sequence-based diversity, we developed a new algorithm based on Rao's quadratic entropy providing an entropy measure by considering distances. Our methods significantly outperforms the original result sets and a clustering-based elimination algorithm, CD-HIT, for various databases, including non-redundant ones. We also evaluate the functional diversity of the result set based on GO terms. Our methods improve the original result set and the CD-HIT method in terms of functional diversification.

Diversity in biological sequences is a novel, and potentially very useful approach. Diversification of sequence similarity search results promise biologists more efficient exploration of the potential functional landscape of the query sequence. By integrating other biological information such as sub-cellular localization and related pathways into diversity measures, diversification may be tailored to specific biological goals.
